# Effect of a Community-Based Holistic Care Package on Physical and Psychosocial Outcomes in People with Lower Limb Disorder Caused by Lymphatic Filariasis, Podoconiosis, and Leprosy in Ethiopia: Results from the EnDPoINT Pilot Cohort Study

**DOI:** 10.4269/ajtmh.21-1180

**Published:** 2022-07-18

**Authors:** Rachael Dellar, Oumer Ali, Mersha Kinfe, Asrat Mengiste, Gail Davey, Stephen Bremner, Maya Semrau, Abebaw Fekadu

**Affiliations:** ^1^ Brighton and Sussex Centre for Global Health Research, Brighton and Sussex Medical School, University of Sussex, Brighton, United Kingdom;; ^2^ Center for Innovative Drug Development and Therapeutic Trials for Africa, Addis Ababa University, Addis Ababa, Ethiopia;; ^3^ School of Public Health, Addis Ababa University, Addis Ababa, Ethiopia;; ^4^ Department of Primary Care and Public Health, Brighton and Sussex Medical School, University of Sussex, Brighton, United Kingdom

## Abstract

Lymphatic filariasis (LF), podoconiosis, and leprosy are highly stigmatized neglected tropical diseases that can cause lower limb swelling and deformity. Simple interventions to support self-care can reduce their physical impacts, but little is known about how to address the psychosocial needs of people living with the diseases, and about how to scale-up morbidity reduction programs. EnDPoINT is a multistage implementation study designed to address these knowledge gaps by developing and evaluating a holistic care package that can be integrated into the Ethiopian health system. This article presents the quantitative results from the EnDPoINT pilot, in which the effectiveness of the care package was assessed in 251 participants from one district in northern Ethiopian using a pre-post design. Reductions 12 months after care package initiation were seen in attacks of acute adenolymphangitis (adjusted odds ratio for attack in last month 0.005; 95% CI 0.001, 0.02; *P* < 0.001), lower limb and foot circumference (mean difference lower limb circumference −2.0 cm; 95% CI −2.3, −1.8; *P* < 0.001; foot circumference −2.3 cm; 95% CI −2.5, −2.0; *P* < 0.001), and lymphedema stage (mean reduction in stage −0.27; 95% CI −0.37, −0.19; *P* < 0.001). Significant improvements were also observed in scores assessing disability, quality-of-life, depression, stigma, discrimination, and social support. This study thus suggests that the EnDPoINT care package is highly effective in reducing morbidity in people living with LF, podoconiosis, and leprosy in northern Ethiopia.

## INTRODUCTION

The neglected tropical diseases (NTDs) lymphatic filariasis (LF), podoconiosis, and leprosy can all cause lower limb lymphedema and consequent pain, swelling, and deformity (Box 1). As highly stigmatized conditions, they have a huge impact on the lives of the people they affect, causing physical disability, reducing economic productivity, and impairing mental well-being.^1^^–^^7^

Morbidity management and disability prevention (MMDP) services have been shown to be effective in mitigating the impacts of all three of these diseases. Several studies, including randomized controlled trials, have demonstrated that encouraging simple self-care measures to promote foot hygiene can reduce limb swelling and improve quality-of-life.^8^^–^^14^ Self-care can also reduce the incidence of the most disabling complication of the lower limb disorders, where the leg becomes acutely red and painful secondary to acute adenolymphangitis (ADL).^8^^,^^9^^,^^11^^–^^13^^,^^15^

Despite the successes of MMDP in study pilot programs, the question remains as to how to integrate wide-scale MMDP services into the often fragile health systems of the resource-limited countries where they are most needed. A key strategy for implementation promoted by the WHO and others is integrating services across diseases that are managed through common strategies: a prime example being the need for simple self-care education in LF, podoconiosis, and leprosy.^16^^,^^17^ However, while a recent proof-of-concept study has shown success with the combined management of these diseases,^14^ again, very little is known about how to scale-up this strategy. There is also a dearth of literature on how to effectively reduce stigma and address the psychosocial needs of people living with the conditions.

The “Excellence in Disability Prevention Integrated across NTDs” (EnDPoINT) implementation study aims to address current knowledge gaps by assessing the integration of a holistic package of care for patients with LF, podoconiosis, and leprosy into the routine Ethiopian healthcare system.^18^ The intervention package will aim to address both the physical and psychosocial needs of people living with the lower limb disorders.

Ethiopia was selected for the EnDPoINT study as it has a pressing need for a sustainable MMDP program for lower limb disorders. Indeed, the development of such a program has been identified as a priority by the Ethiopian Federal Ministry of Health, with Ethiopia home to an estimated 5.6 million people at risk of LF, 1.5 million living with podoconiosis, and 300,000 affected by leprosy.^17^

EnDPoINT is designed in three phases, which correspond to the Medical Research Council’s framework for the assessment of complex interventions^19^: Phase I involves care package development, Phase II package piloting and evaluation in one district, and Phase III rollout and evaluation of the package in several districts. In this article, the results of Phase II are presented: an evaluation of the impact of the pilot EnDPoINT intervention on physical and psychosocial outcomes of patients with LF, podoconiosis, and leprosy in the Guagusa Shikudad district of northwest Ethiopia.

## MATERIALS AND METHODS

### Study design.

The full protocol for the EnDPoINT study has been published elsewhere.^18^ Phase II was designed to assess the care package in one district using a quasi-experimental before-and-after design, wherein physical and psychosocial characteristics were compared between baseline and at 3 and 12 months after initiation of the EnDPoINT care package.

### Study setting.

The study was conducted in the Gusha area in the Guagusa Shikudad district of northwest Ethiopia. This is a rural farming setting with a population of approximately 30,600. The Gusha area consists of five *kebeles* (small administrative units), and each *kebele* has its own health post. A central health center also exists at Gusha.

The setting was chosen due to the lack of other MMDP programs in the area and due to the co-endemicity of LF, podoconiosis, and leprosy, with an estimated combined prevalence of 1.0%.^3^

### Study participants.

All people living with LF, podoconiosis, and leprosy in the Gusha area were invited to consider participation in the study nonrandomly. No formal study size calculation was performed; the aim was to capture all those with lymphedema in the area.

Potential participants were identified from local health records and approached by community-based health professionals known as health extension workers.

Other than the presence of one of the three diseases (as defined by local health records), inclusion criteria were age > 18 years, living in the district for > 6 months, and ability to communicate in the Amharic language. Exclusion criteria were presence of a terminal illness that prevented engagement in the care package and the presence of nodules or wounds that required surgical or specialist management (those in the latter category were appropriately referred). All eligible patients who provided full informed consent were enrolled.

### Study care package.

**Figure 1.**
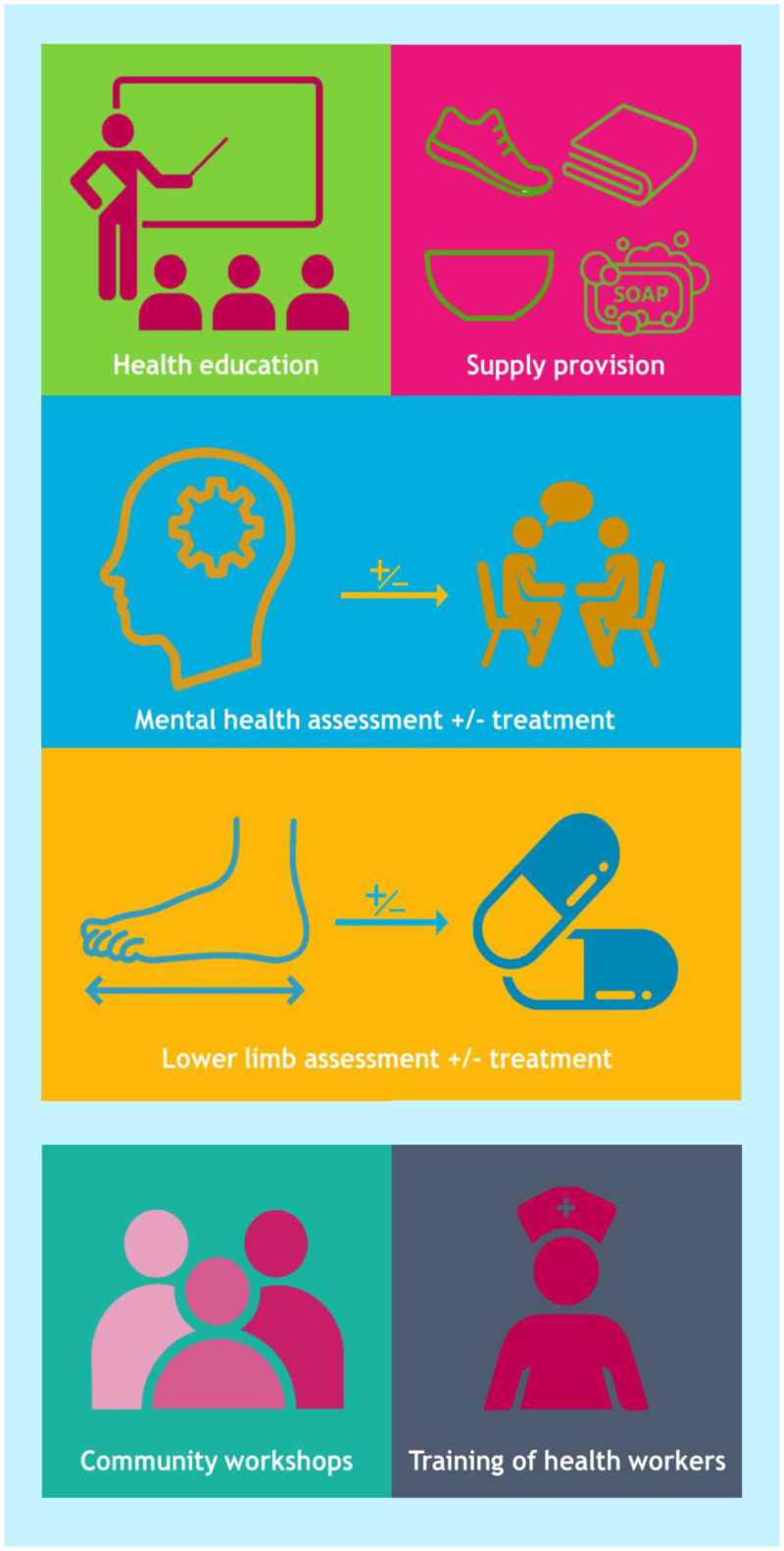
Overview of the EnDPoINT care package. For individuals with lymphedema, the package consists of regular health education sessions that focus on encouraging self-care and improving foot hygiene; supply of custom-made shoes and simple equipment for washing; assessment of mental health, and if required, treatment with antidepressants or counseling; assessment of lower limbs, and if required, treatment with antibiotics or appropriate ointment/bandaging. In the wider community, the package involves community workshops for stigma reduction and training of health professionals. This figure appears in color at www.ajtmh.org.

The study care package consisted of several components and was delivered to participants between August 2019 and October 2020 (Figure 1).

The package was delivered by local healthcare professionals (nurses and health officers) who had received a 5-day theory-based training course in MMDP and mental health. Mental health training was guided by the WHO Mental Health Gap Action Program resources, and also involved a 5-day practical attachment in a hospital that was facilitated by a psychiatrist.^20^

At baseline, healthcare professionals conducted assessments of participants’ lower limbs and mental health. If required, acute treatment was arranged. For lower limb problems, acute treatment options included analgesia, antifungals, and antibiotics. For mental health problems (identified using Patient Health Questionnaire-9 [PHQ-9] as a screening tool and by clinical assessment), counseling, antidepressants, and antipsychotics were available, and follow-up was arranged as necessary.

All participants received counseling and introductory health education sessions delivered by health professionals trained in MMDP. The sessions focused on teaching participants self-care methods to improve foot hygiene such as foot washing and bandaging. A basic package of supplies (wash bowl, soap, salt, Vaseline, and towels) was provided, and participants encouraged to perform the self-care they had learned daily throughout the 12-month study period. At baseline, participants with enlarged feet were also measured for custom-made shoes to be delivered at their 3-month review.

After the baseline assessments and counseling and education sessions, participants met in self-help groups once a fortnight. Every month, these meetings were attended by a healthcare professional who provided further health education and were able to troubleshoot any new reported lower limb or mental health problems. Simple assessments of participants’ limbs were also made at these meetings, and if necessary basic treatments were provided, as described earlier.

Formal assessments of participants’ limbs and mental health were repeated at 3 and 12-months, which provided further opportunity for intervention beyond self-care if required.

In addition to the interventions targeted to the participants, the care package also involved community-level workshops to raise awareness and reduce stigma, and training of healthcare workers and certain community members in MMDP.

Unfortunately, the COVID-19 pandemic impacted the delivery of the planned care package. In April 2020, Ethiopia went into a state of emergency for 5 months, which restricted some study activities. Specifically, health professionals were temporarily not able to attend the monthly health education sessions and attendance at these sessions was reduced. Daily self-care continued throughout and the study and all formal follow-up was completed as planned.

### Study outcomes.

This study was concerned with assessing the effectiveness of the care package on a number of physical and psychosocial outcomes. All outcomes were assessed at baseline and at 3 and 12 months after initiation of the EnDPoINT care package.

#### Physical outcomes.

Physical outcomes included the presence of acute “attack” of ADL in the last month (“attack” defined as leg becoming hot, painful, and more swollen), number of ADL attacks self-reported in the last month, average maximum lower limb circumference (measured at the widest point of the calf and averaged across both legs), average maximum foot circumference (measured at the widest point of the foot and averaged across both feet), presence of wounds or nodules on either leg (nodules being elevated skin lesions > 10 mm that commonly complicate both podoconiosis and LF), and signs of infection on either leg.

Lymphedema stage was also included as a physical outcome measure. The staging system used in this study was developed for podoconiosis, and consists of five stages ranging from 1 (least severe) to 5 (most severe).^21^ The podoconiosis-specific scale was selected due to its high reliability for lymphoedema staging with limited training of assessors. As a “field-friendly” measure with many similarities to more general lymphedema staging methods, it has frequently been used to assess nonpodoconiosis lymphedema. However, it should be acknowledged that it has never been formally assessed for this purpose. An excellent review of the subtle differences in staging for lymphedema is available for the interested reader.^22^

The WHO Disability Assessment Schedule-2.0 (WHODAS-2.0) score was used as an outcome to assess disability.^23^ This is a commonly used tool that has recently been validated in Ethiopia and that has a possible range of 12–60, where higher scores reflect greater disability.^24^

#### Psychosocial outcomes.

The PHQ-9 score was used as a measure of depression.^25^ Total scores for PHQ-9 range from 0 (no depressive symptoms) to 27 (significant depressive symptoms).

Dermatology Life Quality Index (DLQI) scores were used to assess quality-of-life.^26^ The minimum score is 0 (no effect of disease on life) and the maximum is 30 (extremely large effect of disease on life). Importantly, in this study, the DLQI scores reported are likely to be underestimated as one of the 10 questions (“how much has your skin created problems with your partner or any of your close friends or relatives?”) had to be excluded due to an error in data collection; the maximum possible score for this study is thus 27.

To review discrimination, the discrimination section of the Discrimination and Stigma Scale (DISC-12) score was used.^27^ The 22-question discrimination section of the original DISC-12 was modified as described in a previous Ethiopian study by removing two items deemed to not be applicable to the rural Ethiopian setting of the study and two items with low item-factor loading.^28^ Thus, in this study, the possible discrimination scores range from 1to 56, with higher scores reflecting more discrimination.

An outcome to reflect disease-related stigma was adapted from the Internationalized Stigma in Mental Illness (ISMI) scale.^29^ The 29-item ISMI score originally developed for mental illness was adapted by experts in lymphedema to produce an 11-item score relevant to lymphedema. Of note, this score should be interpreted with caution as it has not been formally validated. The range of the score possible in this study is 11 (less stigma) to 44 (more stigma).

Social support was measured using the Oslo-3 social support scale (OSSS).^30^ Total scores from this brief score range from 3 to 14, with higher scores representing better support.

### Data collection and analysis.

All data were collected by trained field staff. Data analysis was performed in Stata IC v. 13.^31^ Mixed effects regression models with a random effect for participant and fixed effect for time-point were used to assess the statistical significance of trends in outcomes before and at 3 and 12 months after initiation of the intervention. All models were adjusted for participant’s sex, age, literacy, relative income, marital status, and whether participants had children or not. These variables were included for adjustment as they were all thought to potentially impact physical or mental health outcomes. Logistic regression was used for dichotomous outcomes, using the mean and variance adapted Gauss–Hermite quadrature as the integration method. Linear regression was used for continuous variables, with robust standard errors estimated for nonnormally distributed variables.

### Ethics.

This research was conducted in accordance with the principles embodied in the Declaration of Helsinki.^32^ Ethical approval was obtained from the Institutional Review Board of the College of Health Sciences, Addis Ababa University, Addis Ababa, Ethiopia (reference 061/18/CDT) and Brighton and Sussex Medical School Research Governance and Ethics Committee, Brighton, United Kingdom (reference ER/BSMS9D79/2). The STROBE checklist for observational studies was used to strengthen the reporting of the study.^33^

## RESULTS

### Sociodemographic characteristics of participants.

A total of 251 people with lower limb disorders caused by LF, podoconiosis, and leprosy were enrolled into the study at baseline. The sociodemographic characteristics of study participants are summarized in Table 1.

**Table 1:** Sociodemographic characteristics of participants

Characteristic	Participants (*N* = 251)
**Age (years); mean (range)**	51.9 (18–88)
**Age category (years); *n* (%)**	
18–24	10 (4.0)
25–34	25 (10.0)
35–44	40 (15.9)
45–54	55 (21.9)
55–64	57 (22.7)
≥ 65	64 (25.5)
**Female sex, *n* (%)**	132 (52.6)
**Rural residence, *n* (%)**	250 (99.6)
**Marital status, *n* (%)**	
Never married	25 (10.0)
Married	170 (67.7)
Divorced	27 (10.8)
Widowed	29 (11.6)
**Have children, *n* (%)**	223 (88.8)
**Number of children, mean (range)**	4.2 (0–10)
**Christian religion, *n* (%)**	251 (100)
**Literacy, *n* (%)**	95 (37.8)
**Received formal education, *n* (%)**	14 (5.6)
**Highest educational achievement for those with formal education, *n*/*N* (%)**	
Primary school, basic (grades 1–4)	7/14 (50.0)
Primary school, general (grades 5–8)	3/14 (21.4)
Beyond primary school	4/14 (28.6)
**Employment status, *n* (%)**	
Salaried	2 (0.8)
Self-used	2 (0.8)
Farming	194 (77.3)
Housework	47 (18.7)
Not working	4 (1.6)
Other	2 (0.8)
**Relative income (self-rated), *n* (%)**	
Very low	40 (15.9)
Low	106 (42.2)
Middle	99 (39.4)
High	6 (2.4)

The mean age of participants was 51.9 years, with an age range of 18–88 years. Female participants accounted for 52.6% of the total (132/251).

The vast majority of participants lived in rural areas (99.6%; 250/251). Literacy levels were low, with only 37.8% (95/251) able to read or write, and only 5.6% (14/251) reporting having received any formal education.

Most participants worked in farming (77.3%; 194/251). Relative to others in their community, 15.9% (40/251) rated their income as very low, 42.2% (106/251) as low, 39.4% (99/251) as middle, and 2.4% (6/251) as high.

All participants identified as Christian, 170 (67.7%) were married, and 223 (88.8%) had children.

Participants were followed up at 3 months and 12 months after the start of the intervention. Twenty-six participants (10.4%) were lost to follow-up between baseline and 12 months. No sociodemographic characteristics were significantly associated with loss-to-follow-up.

### Physical characteristics of participants at baseline.

Of those enrolled into the study, 98.0% (246/251) had LF or podoconiosis and 2.0% had leprosy. At baseline, only 21 participants (8.4%) reported to have been receiving treatment of their lower limb disorder, and physical characteristics were generally poor (Table 2).

**Table 2:** Physical and psychosocial characteristics of participants at baseline and after 3 and 12 months of receiving the study care package

	Baseline *N* = 251	After intervention	
		3 months	12 months
		*N* = 234	*N* = 225
**Physical characteristics**			
**Diagnosis, *n* (%)**			
Lymphatic filariasis/podoconiosis	246 (98.0)	231 (98.7)	221 (98.2)
Leprosy	5 (2.0)	3 (1.3)	4 (1.8)
**Currently receiving treatment, *n* (%)**	21 (8.4)	234 (100)	225 (100)
**Maximum lower limb circumference, mean (SD)**	26.8 (4.0)	25.0 (4.0)	24.9 (3.7)
**Maximum foot circumference, mean (SD)**	27.8 (2.9)	25.7 (2.8)	25.5 (2.6)
**Presence of wounds, *n* (%)**	7 (2.8)	5 (2.1)	2 (0.9)
**Presence of nodules, *n* (%)**	49 (19.5)	16 (6.8)	3 (1.3)
**Signs of infection, *n* (%)**	33 (13.1)	12 (5.1)	4 (1.8)
**Lymphedema stage, mean (SD)**	2.7 (1.5)	2.3 (1.4)	2.4 (1.4)
**Attack of ADL in last month, *n* (%)**	200 (79.7)	40 (17.1)	17 (7.6)
**Number of ADL attacks in last month, mean (SD)**	2.2 (2.5)	0.5 (1.5)	0.1 (0.5)
Disability (WHODAS-2.0), mean (SD)*	29.6 (8.8)	19.2 (6.7)	18.8 (6.5)
**Psychosocial characteristics**			
**Quality-of-life score (adjusted DLQI), mean (SD)**†	10.9 (4.5)	4.0 (4.3)	3.8 (4.1)
**Depressive symptom score (PHQ-9), mean (SD)**‡	10.0 (5.2)	5.0 (4.9)	5.1 (4.9)
**Presence of at least moderate depressive symptoms (PHQ-9 ≥ 10), *n* (%)**	119 (47.4)	60 (25.6)	37 (16.4)
**Disease-related stigma score (adapted ISMI), mean (SD)**§	27.7 (6.8)	23.9 (8.2)	23.9 (6.4)
**Diseased-related discrimination score (adapted DISC-12), mean (SD)** ^‖^	7.6 (7.6)	5.7 (6.6)	4.7 (6.0)
**Social support score (OSSS), mean (SD)** ^¶^	7.2 (2.9)	7.6 (2.8)	7.8 (2.9)
**Social support category (OSSS** **), *n* (%)**			
Poor (3–8)	164 (65.3)	153 (65.3)	140 (62.2)
Moderate (9–11)	69 (27.5)	59 (25.2)	55 (24.4)
Strong (12–14)	18 (7.2)	22 (0.9)	31 (13.2)

ADL = acute adenolymphangitis; DISC-12 = discrimination score-12; DLQI = Dermatology Life Quality Index; ISMI = internalized stigma related to mental illness; OSSS = Oslo social support score; PHQ-9 = Patient Health Questionnaire-9; WHODAS-2.0 = World Health Organization Disability Assessment Schedule-2.0.

* Higher scores reflect increased disability; possible range 12–60.

† Higher scores reflect worse quality-of-life; possible range 0–27.

‡ Higher scores reflect a higher frequency of depressive symptoms; possible range 0–27.

§ Higher scores reflect increased experience of stigma; possible range 11–44.

‖ Higher scores reflect increased experience of discrimination; possible range 1–56.

¶ Higher scores reflect increased social support; possible range 3–14.

Two hundred participants (79.7%) had suffered from an attack of ADL in the last month. Signs of infection were observed in 13.1% (33/251). Nodules and wounds were observed in 19.5% (49/251) and 2.8% (7/251), respectively. Mean maximum lower limb circumference was 26.8 cm (SD 4 cm) and mean maximum foot circumference was 27.8 cm (SD 2.9 cm).

Mean lymphedema in podoconiosis stage was 2.7 (SD 1.5), with stage 2 representing below-knee swelling not reversible overnight with or without nodules below ankle, and stage 3 below-knee swelling not reversible overnight with nodules above ankle. The mean WHODAS-2.0 score, a measure of disability, was high at 29.6 (SD 8.8).

### Physical characteristics of participants after 3 and 12 months of care package intervention.

All physical measures improved 3 and 12 months after initiating the study care package (Table 2).

The proportion of participants having an acute attack of ADL in the last month decreased significantly from 79.7% at baseline, to 17.1% at 3 months and 7.6% at 12 months. In a mixed effects logistic regression model adjusted for sociodemographic characteristics (Table 3), the adjusted odds ratio (aOR) for having any ADL attack in the last month was 0.03 at 3 months (95% CI 0.01, 0.07; *P* < 0.001) and 0.005 at 12 months (95% CI 0.001, 0.02; *P* < 0.001).

**Table 3:** Assessing the impact of the study care package on physical and psychosocial outcomes after 3 and 12 months

	After 3 months of intervention			After 12 months of intervention		
Physical outcomes (continuous): linear regression	Mean difference in outcome compared with baseline	95% CI	*P* value	Mean difference in outcome compared with baseline	95% CI	*P* value
**Mean maximum lower limb circumference (cm)**	−1.96	−2.29, −1.64	< 0.001	−2.02	−2.26, −1.77	< 0.001
**Mean maximum foot circumference (cm)**	−2.21	−2.46, −1.96	< 0.001	−2.28	−2.53, −2.04	< 0.001
**Mean ADL attacks in last month**	−1.71	−2.10, −1.33	< 0.001	−2.12	−2.45, −1.79	< 0.001
**Disease stage**	−0.32	−0.43, −0.22	< 0.001	−0.27	−0.37, −0.19	< 0.001
Disability score (based on WHODAS 2.0)*	−10.22	−11.42, −9.02	< 0.001	−10.97	−12.08, −9.87	< 0.001

Physical outcomes (dichotomous): logistic regression	aOR	95% CI	*P* value	aOR	95% CI	*P* value
**ADL attack in last month (yes vs. no)**	0.03	0.01, 0.07	< 0.001	0.005	0.001, 0.02	< 0.001
**Presence of wounds (yes vs. no)**	1.39	0.19, 10.23	0.744	0.16	0.02, 1.79	0.138
**Presence of nodules (yes vs. no)**	0.13	0.05, 0.39	< 0.001	0.02	0.001, 0.28	0.005
**Signs of infection (yes vs. no)**	0.17	0.06, 0.47	0.001	0.08	0.02, 0.27	< 0.001

Psychosocial outcomes (continuous): linear regression	Mean difference in outcome compared with baseline	95% CI	*P* value	Mean difference in outcome compared with baseline	95% CI	*P* value
**Quality-of-life score (adjusted DLQI)**†	−6.93	−7.64, −6.22	< 0.001	−7.18	−7.88, −6.48	< 0.001
**Depressive symptom score (PHQ-9)**‡	−4.96	−5.78, −4.14	< 0.001	−5.06	−5.85, −4.26	< 0.001
**Disease-related stigma score (adapted ISMI)**§	−3.79	−4.99, −2.59	< 0.001	−3.87	−4.86, −2.87	< 0.001
**Disease-related discrimination score (adapted DISC-12)** ^‖^	−2.22	−3.42, −1.04	< 0.001	−3.22	−4.31, −2.12	< 0.001
**Social support score (OSSS)**¶	0.36	−0.08, 0.80	0.106	0.61	0.18, 1.03	0.005

aOR = adjusted odds ratio; WHODAS-2.0 = World Health Organization Disability Assessment Schedule-2.0; ADL = acute adenolymphangitis; DLQI = Dermatology Life Quality Index; PHQ-9 = Patient Health Questionnaire-9; ISMI = internalized stigma related to mental illness; DISC-12 = discrimination score-12; OSSS = Oslo social support score. Mixed-effect linear and logistic regression modeling with random effect for participant and fixed effect for time-point; adjusted for participant age, sex, literacy, marital status, relative income rating, and presence of children.

* Higher scores reflect increased disability; possible range 12–60.

† Higher scores reflect worse quality-of-life; possible range 0–27.

‡ Higher scores reflect a higher frequency of depressive symptoms; possible range 0–27.

§ Higher scores reflect increased experience of stigma; possible range 11–44.

‖ Higher scores reflect increased experience of discrimination; possible range 1–56.

¶ Higher scores reflect increased social support; possible range 3–14.

The mean *number* of ADL attacks in the last month decreased significantly from 2.2 (SD 2.5) at baseline to 0.5 (SD 1.5) at 3 months (mean reduction −1.71, 95% −2.10, −1.33; *P* < 0.001). At 12 months, the mean number of ADL attacks in the last month was 0.1 (SD 0.5), a mean reduction of −2.12 compared with baseline (95% CI −2.45, −1.79; *P* < 0.001).

Signs of infection were observed in 13.1% of participants at baseline, compared with 5.1% at 3 months, and 1.8% at 12 months. These reductions in infection yielded significant aORs of 0.17 at 3 months (95% CI 0.06, 0.47; *P* = 0.001) and 0.08 at 12 months (95% CI 0.02, 0.27; *P* < 0.001). The proportion of participants with nodules was also significantly reduced across time-points (19.5% at baseline versus 6.8% at 3 months versus 1.3% at 12 months; aOR [3 months] 0.13; 95% CI 0.05, 0.39; *P* < 0.001; aOR [12 months] 0.02; 95% CI 0.001, 0.28; *P* = 0.005). Wounds were observed less frequently after the intervention (2.8% at baseline versus 2.1% at 3 months versus 0.9% at 12 months), however, these changes were not significant (aOR [3 months] 1.39; CI 0.19, 10.23; *P* = 0.744; aOR [12 months] 0.16; 95% CI 0.02, 1.79; *P* = 0.138).

Average maximum lower limb and foot circumference were both found to be significantly reduced after both 3 and 12 months of follow-up in an adjusted mixed-effects linear regression model. After 12 months, the adjusted mean reduction in lower limb circumference was 2.0 cm (95% CI 1.8 cm, 2.3 cm; *P* < 0.001), and in foot circumference was 2.3 cm (95% CI 2.0 cm, 2.5 cm; *P* < 0.001). Disease stage also showed a modest but significant reduction after care package rollout (adjusted mean reduction at 12 months 0.3; 95% CI 0.2, 0.4; *P* < 0.001).

Disability (WHODAS 2.0) scores improved from 29.6 at baseline to 19.2 at 3 months and 18.8 at 12 months. The adjusted mean reduction in disability scores at 3 months and 12 months were 10.2 (95% CI 9.0, 11.4; *P* < 0.001) and 11.0 (95% CI 9.9, 12.1; *P* < 0.001), respectively.

### Psychosocial characteristics of participants at baseline.

Similar to physical characteristics, psychosocial characteristics of study participants at baseline were generally poor (Table 2).

There was evidence that the lower limb disorders the participants suffered from had at least a moderate effect on their quality-of-life at baseline, with a mean adjusted DLQI score of 10.9 (SD 4.5). Further, the majority of patients (86.5%; 217/251) were found to have at least mild depressive symptoms (PHQ-9 score ≥ 5), and a large proportion (47.4%; 119/251) had at least moderate depression. The mean PHQ-9 score at baseline was 10.0 (SD 5.2).

The mean stigma score (adapted ISMI) at baseline was 27.7 (SD 6.8; possible range 11–44; greater scores represent greater stigma). The mean baseline discrimination (adapted DISC-12) score was 7.6 (SD 7.6; possible range 1–56; greater scores represent greater discrimination).

Using the OSSS, at baseline, the majority of participants were found to have poor social support (65.3%; 164/251). The mean OSSS was 7.2 (SD 2.9), where the possible range is 3–14 and higher scores represent increased social support.

### Psychosocial characteristics of participants after 3 and 12 months of care package intervention.

Psychosocial outcomes were reassessed 3 and 12 months after initiation of the care package. As for physical characteristics, there was a trend to improvement in all outcomes.

Quality-of-life (adjusted DLQI) scores improved from 10.9 at baseline to 4.0 at 3 months and 3.8 at 12 months. In an adjusted mixed effects linear regression model (Table 3), the mean improvement in quality-of-life score after 3 months was 6.9 (95% CI 6.2, 7.6; *P* < 0.001), and after 12 months was 7.2 (95% CI 6.5, 7.9; *P* < 0.001).

Depressive symptom (PHQ-9) scores were also found to be significantly improved. From baseline to 3 months, scores decreased by an adjusted mean of 5.0 (95% CI 4.1, 5.8; *P* < 0.001) and from baseline to 12 months by 5.1 (95% CI 4.3, 5.9; *P* < 0.001).

Levels of self-reported stigma faced by participants decreased, with stigma scores (adapted ISMI) falling significantly by a mean of 3.8 (95% CI 2.6, 5.0; *P* < 0.001) between baseline and 3 months and by 3.9 (95% CI 2.9, 4.9; *P* < 0.001) between baseline and 12 months.

Discrimination scores (adapted DISC-12) similarly decreased significantly from 7.6 at baseline to 5.7 at 3 months and 4.7 at 12 months. The mean improvement in score after 12 months of the care package was 3.2 (95% CI 2.1, 4.3; *P* < 0.001).

Social support scores (OSSS) were 7.2 at baseline, 7.6 at 3 months, and 7.8 at 12 months. Although there was a modest trend to improvement at 3 months, this was not significant at this time-point (adjusted mean 0.4; 95% CI −0.1, 0.8; *P* = 0.11). From baseline to 12 months, however, the trend gained significance (adjusted mean 0.6; 95% CI 0.2, 1.0; *P* = 0.005).

## DISCUSSION

This study found that over the time period of the EnDPoINT intervention there were significant improvements in a range of physical and psychosocial outcomes for patients with lymphedema caused by LF, podoconiosis, and leprosy in northern Ethiopia.

Substantial improvements in lower limb disease were noted after both 3 and 12 months of implementing the EnDPoINT care package. Significant reductions were seen in frequency of attacks of ADL, lower limb and foot size, the presence of nodules, signs of infection, lymphedema stage, and overall level of disability. Wounds were also less frequent after the intervention, although this reduction was not statistically significant.

The psychosocial benefits noted over the study period included significantly improved quality-of-life, reduced levels of depression, and reduced experiences of stigma and discrimination. After 12 months, a modest but significant increase in social support was also noted. Interestingly, most of the improvements in psychosocial outcomes were observed between baseline and 3 months. This might suggest that improvements can be made to mental well-being over even a very short time frame. However, another possible explanation for this trend could be the reduced attendance at support meetings that resulted from COVID-19 restrictions. Results from Phase III of EnDPoINT are eagerly awaited to bring clarity to this issue.

**Figure 2.**
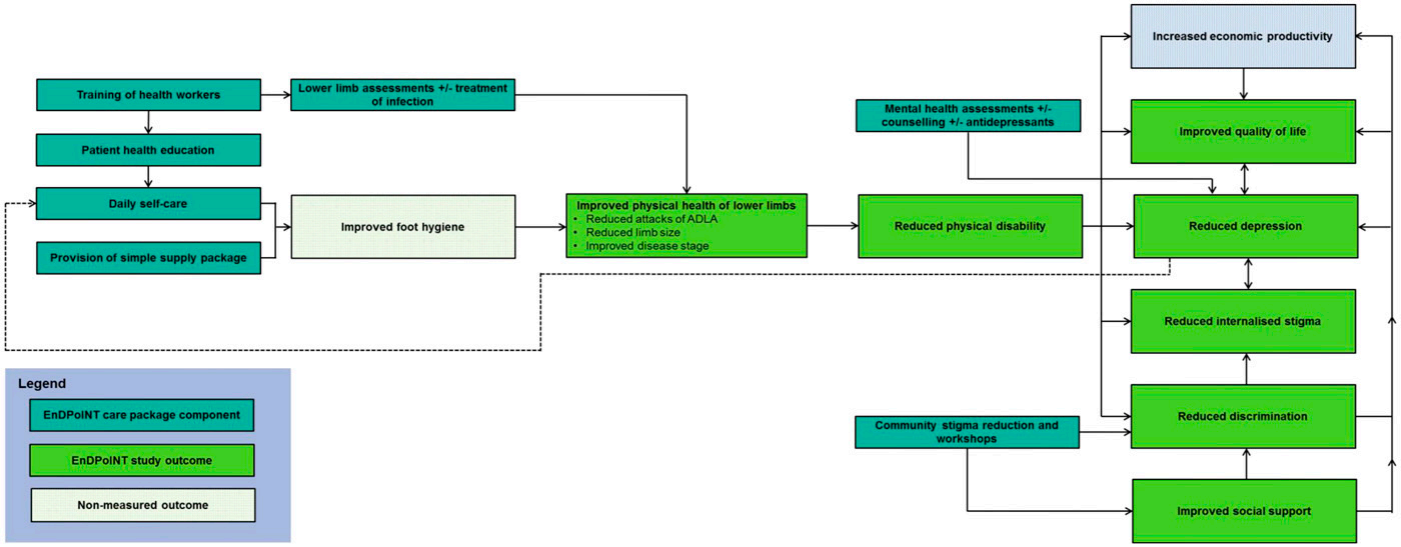
Theoretical framework for understanding the impact of the various components of the EnDPoINT intervention on study outcomes. This figure appears in color at www.ajtmh.org.

As this study was not randomized, any significant trends seen over the study time frame can only ever be *assumed* to be a result of the intervention itself. However, theoretical plausibility and previous literature support a causal link between the EnDPoINT care package and the improvements in physical and psychosocial outcomes.^8^^–^^13^ Physical benefits are presumed to result from improved health education, daily lower limb self-care, and provision of a simple supply package (Figure 2). The positive impact on psychosocial outcomes is proposed to stem both directly from the components of the intervention designed to address low mood and reduce community-level stigma, and indirectly through improvements in clinical disease and disability (Figure 2).

Notably, while challenging to compare, the impact of the EnDPoINT intervention on the frequency of ADL attacks appears greater than that reported in other studies, including a randomized controlled trial conducted in a similar Ethiopian setting.^8^^,^^9^^,^^11^^,^^13^ The GoLBeT study aimed to assess the impact of a foot care package in people with podoconiosis and found that ADL incidence was approximately 20% lower in the intervention arm compared with the control arm.^8^ In comparison, mean ADL episodes self-reported in the last month in this study improved from 2.2 at baseline to 0.1 at follow-up, an approximately 95% decrease.

The apparent differences in effect size on ADL frequency between this study and GoLBeT may be due to a number of factors. First, as previously discussed, this study was nonrandomized, so it is possible there may be other factors that have affected ADL frequency outside of the intervention itself. Second, the patient cohorts in the two studies were different, as GoLBeT only recruited people with at least stage 2 podoconiosis, whereas this study recruited those with LF, podoconiosis, and leprosy of any disease stage. Third, the way ADL frequency was measured differed. GoLBeT gave patients diaries to record ADL episodes and reviewed these quarterly, whereas EnDPoINT relied on patients to recall the number of ADL episodes they had had in the last month. A previous study has shown that people with lymphoedema recorded ADL symptoms more frequently in diaries than were reported in recall, and so it may be that there was an underreporting of ADL episodes in EnDPoINT relative to GoLBeT.^33^ Finally, it may be that the difference in effect sizes is a result of the EnDPoINT intervention truly being more effective in reducing ADL incidence. While the care packages for encouraging foot hygiene are similar, it is plausible that addressing the psychosocial needs of people with the diseases could have resulted in improved self-motivation and social support for performing daily self-care. Clearly, this will be an important area for future research, including quantitative studies to assess adherence to self-care and its determinants, and qualitative studies to understand the potential impact of improved mental health on lower limb care.

Effect sizes in other physical outcomes, including reductions in lower limb circumference, were more comparable across studies in other Ethiopian settings.^8^^,^^10^^,^^14^

In terms of psychosocial outcomes, it is challenging to compare the results presented here with previous work, as there is limited research in this area, and measurement often depends on scoring scales that vary between studies. Moreover, beyond nonrandomization, an important further limitation of this study is in the assessment of concepts such as stigma and quality-of-life by scales, which although are mostly well validated are unlikely to capture the full complexity of the outcomes they aim to measure. Further qualitative work from EnDPoINT is awaited to understand the impact of the intervention on psychosocial outcomes in more depth. Qualitative work will also be important in reviewing the acceptability of integration of care across diseases.

This study is also limited by the small sample size from those with leprosy, which reflects the relatively low prevalence of this condition in the study setting. A larger sample is needed to ensure the results presented herein are applicable to this population.

Further, the effects of the intervention on lymphedema stage should be viewed with caution, as a lymphedema staging system designed for patients with podoconiosis was used in patients with lymphedema from other causes. The measure was chosen to be “field-friendly” in a population where care was integrated across diseases, but has never been formally validated outside of podoconiosis.

Nevertheless, in spite of the discussed limitations and need for future work, this pilot study provides consistent evidence to support the fact that the EnDPoINT intervention is effective in improving the physical and psychosocial health of people living with LF, podoconiosis, and leprosy in northern Ethiopia. The results of the evaluation of the care package across several districts in Phase III of EnDPoINT are eagerly anticipated to provide further validation of these results and also important information on the cost-effectiveness and scalability of integrated MMDP services in the Ethiopian setting.

Box 1 **The neglected tropical diseases lymphatic filariasis, podoconiosis, and leprosy**

**Lymphatic filariasis** is caused by infection with filarial parasites transmitted to humans via mosquitoes. The parasites can cause damage to the lymphatic system, resulting in the buildup of fluid in the lower limbs (lymphedema) and/or scrotum (hydrocele).

**Podoconiosis** is a form of lymphedema and lower leg deformity caused by exposure to irritant volcanic soils. It typically affects genetically susceptible individuals living in highland areas who do not wear shoes.

**Leprosy** is a chronic infectious disease caused by the bacterium *Mycobacterium leprae*. It mainly affects the skin, peripheral nerves, upper respiratory tract, and eyes. Untreated leprosy can cause significant skin lesions and deformity.
